# Identifying Evidence-Informed Physical Activity Apps: Content Analysis

**DOI:** 10.2196/10314

**Published:** 2018-12-18

**Authors:** Mihiretu Kebede, Berit Steenbock, Stefanie Maria Helmer, Janna Sill, Tobias Möllers, Claudia R Pischke

**Affiliations:** 1 Prevention and Evaluation Leibniz Institute for Prevention Research and Epidemiology – BIPS Bremen Germany; 2 Health Sciences University of Bremen Bremen Germany; 3 Institute of Public Health University of Gondar Gondar Ethiopia; 4 Center of Clinical Psychology and Rehabilitation University of Bremen Bremen Germany; 5 Institute for Social Medicine, Epidemiology, and Health Economics Charité – Universitätsmedizin Berlin, corporate member of Freie Universität Berlin Humboldt-Universität zu Berlin, and Berlin Institute of Health Berlin Germany; 6 Network Aging Research University of Heidelberg Heidelberg Germany; 7 Institute of Medical Sociology Centre for Health and Society, Medical Faculty University of Duesseldorf Duesseldorf Germany

**Keywords:** guidelines, mHealth, mobile apps, physical activity, mobile phone

## Abstract

**Background:**

Regular moderate to vigorous physical activity is essential for maintaining health and preventing the onset of chronic diseases. Both global rates of smartphone ownership and the market for physical activity and fitness apps have grown rapidly in recent years. The use of physical activity and fitness apps may assist the general population in reaching evidence-based physical activity recommendations. However, it remains unclear whether there are evidence-informed physical activity apps and whether behavior change techniques (BCTs) previously identified as effective for physical activity promotion are used in these apps.

**Objective:**

This study aimed to identify English and German evidence-informed physical activity apps and BCT employment in those apps.

**Methods:**

We identified apps in a systematic search using 25 predefined search terms in the Google Play Store. Two reviewers independently screened the descriptions of apps and screenshots applying predefined inclusion and exclusion criteria. Apps were included if (1) their description contained information about physical activity promotion; (2) they were in English or German; (3) physical activity recommendations of the World Health Organization or the American College of Sports Medicine were mentioned; and (4) any kind of objective physical activity measurement was included. Two researchers downloaded and tested apps matching the inclusion criteria for 2 weeks and coded their content using the Behavioral Change Technique Taxonomy v1 (BCTTv1).

**Results:**

The initial screening in the Google Play Store yielded 6018 apps, 4108 of which were not focused on physical activity and were not in German or English. The descriptions of 1216 apps were further screened for eligibility. Duplicate apps and light versions (n=694) and those with no objective measurement of physical activity, requiring additional equipment, or not outlining any physical activity guideline in their description (n=1184) were excluded. Of the remaining 32 apps, 4 were no longer available at the time of the download. Hence, 28 apps were downloaded and tested; of these apps, 14 did not contain any physical activity guideline as an app feature, despite mentioning it in the description, 5 had technical problems, and 3 did not provide objective physical activity measurement. Thus, 6 were included in the final analyses. Of 93 individual BCTs of the BCTTv1, on average, 9 (SD 5) were identified in these apps. Of 16 hierarchical clusters, on average, 5 (SD 3) were addressed. Only BCTs of the 2 hierarchical clusters “goals and planning” and “feedback and monitoring” were identified in all apps.

**Conclusions:**

Despite the availability of several thousand physical activity and fitness apps for Android platforms, very few addressed evidence-based physical activity guidelines and provided objective physical activity measurement. Furthermore, available descriptions did not accurately reflect the app content and only a few evidence-informed physical activity apps incorporated several BCTs. Future apps should address evidence-based physical activity guidelines and a greater scope of BCTs to further increase their potential impact for physical activity promotion.

## Introduction

Smartphone ownership among adults, including older adults, has rapidly increased worldwide [1]. In Germany, 97% of adults aged 30-49 years, 88% of adults aged 50-64 years, and 41% of those aged ≥65 years reported owning and using a smartphone in 2017 [2]. In tandem with the peak of smartphone ownership, there is an increase in consumer interest in physical activity (PA) measurement assisting individuals in recording their day-to-day activities. The results of a population-based survey conducted in Germany suggested that among German smartphone users (n=4144; age 57 years, SD 14), approximately 21% use health apps to change health behavior, including PA, or to reach certain health outcomes, such as weight loss [3]. Although this analysis is not based on a representative sample, these findings indicate that apps may represent an important vehicle for implementing population-based strategies aimed at health behavior change, including the promotion of regular PA in Germany. This takes on added significance considering the notable demographic change in Germany. Compared with other European countries, Germany is faced with an aging population [4]. Hence, the use of health and fitness apps may facilitate the uptake and maintenance of health and PA behavior [5] and may contribute to healthy aging. The use of such apps may assist older adults in maintaining muscular and cardiorespiratory fitness and bone and functional health [6-8].

In 2015, the Preventive Health Care Act was passed in Germany, which mandates health insurances and long-term insurance funds to invest >500 million euros in health promotion and primary prevention in the coming years [9]. As a result, health insurances have increased their efforts to offer apps for health promotion to their clients. So far, apps for stress reduction, smoking cessation, and making dietary changes have been made available to insurance holders [10]. In some cases, insurance agencies in Germany have even developed apps themselves (eg, fit mit AOK, Generali Vitality, and BARMER App FIT2GO) [10]. Similarly, the market for commercially available health and fitness apps is booming. In 2016, approximately 259,000 mobile health (mHealth) apps (ie, those listed in the medical and health and fitness app category of an app store) were available in major app stores. Google Play (Android) currently displays 97,345 mHealth apps, including apps from both health and fitness and medical categories [11]. It is estimated that in 2020, approximately 2.6 billion app users will have downloaded mHealth apps at least once and 551 million of these app users will actively use the apps [12].

However, it is unclear whether the apps recommended by health insurances or those commercially available are based on existing evidence stemming from research identifying effective intervention components or mechanisms for health behavior change. A growing body of research is examining whether the content, particularly that of PA apps, is based on current evidence on the underlying mechanisms for behavior change. This research suggests that only a few PA apps are evidence informed and address current guidelines for aerobic activity and strength and resistance and flexibility training [7,13,14]. This is a major shortcoming considering that health benefits associated with PA can only be obtained when these recommendations are reached and that particularly older adults (age≥60 years) rarely meet these recommendations [15]. In Germany, only 22% of adults aged ≥45 years meet the current PA recommendations [16]. Thus, making information on PA recommendations salient in PA and fitness apps or designing features around facilitating weekly moderate PA of 150 minutes recommended by the World Health Organization (WHO) are necessary steps to support particularly older users in starting or developing a PA routine [13,14].

In addition, it is unknown which behavior change techniques (BCTs) are used in apps and whether their use is associated with increased behavior change among users. Harries et al [17] suggested that feedback on a person’s personal PA level is itself sufficient to prompt increased walking; in their study, participants who wore an always-on, accelerometer-based smartphone app experienced a substantial increase in walking. In addition, several content analyses have been conducted, predominantly in the Netherlands and the United Kingdom, to identify active components of various types of apps, including PA apps [18,19]. Several content analyses used the Behavioral Change Technique Taxonomy v1 (BCTTv1), a comprehensive and reliable tool for assisting researchers in retrospectively identifying active components of interventions, particularly behavioral interventions. It includes 93 BCTs considered to be effective for behavior change and 16 hierarchical clusters [20]. Middelweerd et al [19] analyzed PA apps, using an earlier taxonomy developed by Michie et al [21], and found that, on average, 5 (range, 2-8) of 23 possible BCTs were used in the reviewed apps. The most frequently used BCTs were “feedback on performance” and “goal setting”, whereas other BCTs of the taxonomy were not identified. In another content analysis, apps for medication adherence were examined using the BCTTv1 [22]. Here, the number of BCTs contained in an app ranged from 0 to 7 out of 96 possible BCTs, and the most commonly used BCTs were “action planning” and “prompt/cues,” which were included in 96% (160/166) of the total of 166 medication adherence apps investigated [22].

In sum, there are many PA and fitness apps commercially available, as well as an increasing number of apps made available by health insurances. However, there is still a lack of research on whether these apps are based on evidence-based PA guidelines and which BCTs are employed. To date, no content analysis evaluating the entire range of BCTs for evidence-informed PA (EIPA) apps with objective PA tracking available on the German market has been published. This is a major shortcoming considering the increased focus on population-based strategies for PA promotion for primary prevention in Germany owing to the Preventive Health Care Act and the need for low-threshold electronic health (eHealth) interventions, including PA apps, which can assist the general population in increasing PA. Hence, this study aims to identify EIPA apps and BCTs employed in both German and English PA and fitness apps, using the BCTTv1 taxonomy.

## Methods

### Definition of Evidence-Informed Physical Activity Apps

The global PA recommendation of the WHO for adults is to engage in 150 minutes of moderate-intensity aerobic PA throughout the week or at least 75 minutes of vigorous-intensity aerobic PA or an equivalent combination of moderate-intensity and vigorous-intensity activity [8]. Furthermore, aerobic activity should be performed in bouts of at least 10 minutes, and whole-body strength training activities for major muscle groups on at least 2 days per week are recommended. The American College of Sports Medicine (ACSM) outlines that at least 10,000 steps per day are needed for adults [7]. These are the two most commonly used guidelines for designing evidence-based PA interventions, including eHealth interventions for PA promotion (Eysenbach defines eHealth as an “intersection of medical informatics, public health and business, referring to health services and information delivered or enhanced through the internet and related technologies” (pg 1) [23]). Therefore, a PA app designed following any of these guidelines is considered as an EIPA app [14].

### Identification of Physical Activity and Fitness Apps

Apps were identified in a comprehensive systematic search in the Google Play Store. The search took place between August 3 and October 6, 2015. We used 25 search terms in German and English to search across all categories in the Google Play Store (ie, Bewegung, Sport, Aktivität, Übungen, Training, Laufen, Gehen, Joggen, Sportliche Aktivität, Fitness, Gleichgewicht, Kräftigung, move, sports, exercise, activity, exercises, workout, walk, run, step, jogging, physical activity, balance, and strengthening). Two reviewers independently screened all results available in the Google Play Store for each single search term during the day of the search. In cases where searches were performed on different days and there were fewer or more apps because of updates, only results of the later search were included.

### Screening Procedure

Because of the comprehensiveness of our search, we divided the screening procedure into the following three different steps. The first step was to identify PA and fitness apps; each search term was entered in the Google Play Store. The names and descriptions of the apps were reviewed based on *a priori* defined inclusion and exclusion criteria. Apps were included if the app description contained information about PA promotion and if they were in English or German. Conversely, they were excluded if the content was focused on topics, such as allergies, babies, nutrition, hypnosis, smoking, pregnancy, and stress, or if they were in a language other than English and German. The name, description, number of installations, and the price of the included apps were extracted into an excel sheet (Multimedia Appendix 1). In case of any discrepancies between the 2 reviewers while applying the inclusion and exclusion criteria, the consensus was reached after discussing with a third reviewer. In the second step, duplicates of apps were removed manually. In the third step, app descriptions were screened, according to the following additional inclusion and exclusion criteria: Inclusion criteria—(1) the app description contained any of the recommendations of the ACSM or the WHO and (2) the app description contained information about an objective assessment of PA (eg, step count, distance in kilometers, or minutes of being active measured through mobile phone’s built-in acceleration sensor or global positioning system); exclusion criteria—(1) a specific group other than the user was targeted (eg, gym owners); (2) the app only focused on training of particular areas of the body and did not target the whole body; (3) necessitated external devices for use (eg, heart rate monitor and accelerometer-based activity monitor). If a full and light version of the same app was available, the light version was excluded. When necessary, we paid for the full version of the app.

### Testing Phase of Evidence-Informed Physical Activity Apps and Behavioral Change Technique Rating Procedure

Apps that met all inclusion criteria were downloaded, installed, and tested on different smartphones running Android operating systems (Samsung Galaxy S6 Edge and Samsung Galaxy S5) by 5 raters from February to May 2017. The content evaluation of the apps was based on the BCTTv1, and BCTs were independently identified by 2 trained raters using the taxonomy [20]. The raters ran all apps for at least 2 weeks on their smartphone to check all features of the apps and extract data on app characteristics (eg, user rating, download rates, and language) and additional functionalities. If an app outlined PA recommendations in the description but none of the recommendations were found in the features of the app during the 2-week testing phase, the app was excluded from the content analysis. After the end of the testing period, the 2 raters independently coded BCTs for each of the remaining EIPA apps. Of note, the results of this study are solely based on the content of each EIPA app. No additional information about the apps was collected from websites of the developers. The information collected and the BCTs coded by the 2 raters were discussed with a third researcher to solve any discrepancies.

### Behavioral Change Technique Taxonomy v1

The BCTTv1 is a tool for assisting researchers in retrospectively identifying effective components of behavioral interventions, including eHealth interventions such as Web-based interventions or smartphone apps. It includes 93 BCTs considered to be effective for behavior change, which are organized into 16 hierarchical clusters [20]. The BCTTv1 has been validated and is used to design and retrospectively evaluate the effects of behavioral health interventions [24].

### Data Analysis

We calculated the interrater reliability between the 2 raters using the commonly used interrater agreement indices: Cohen kappa and prevalence-adjusted and bias-adjusted kappa. Descriptive statistics were used to analyze the number of BCTs addressed in the examined apps. Data analysis was performed using IBM SPSS Statistics for Windows, version 24.0 (IBM Corp).

## Results

**Figure 1:**
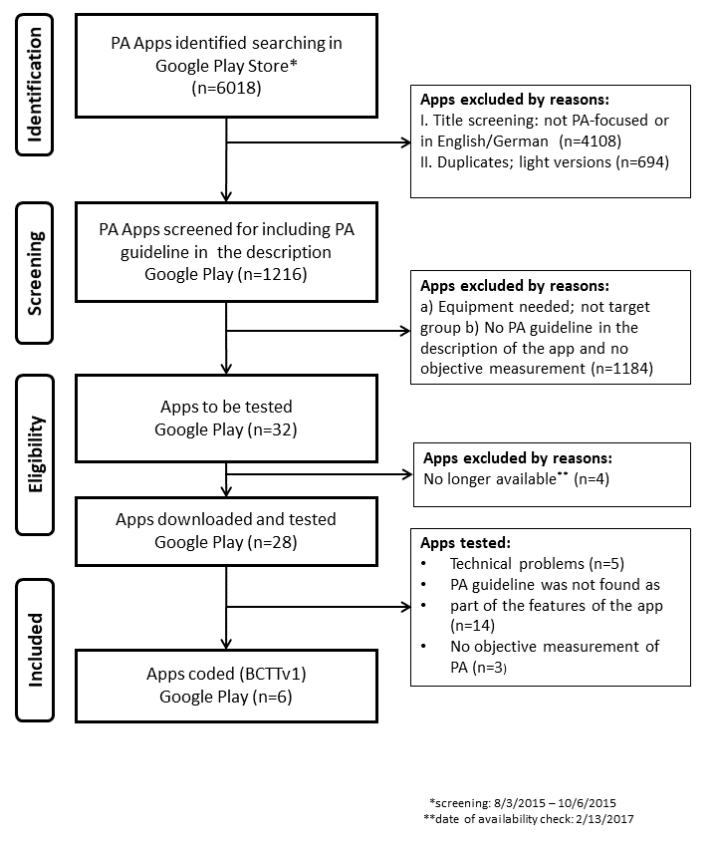
Overview of steps for selecting physical activity (PA) and fitness apps. BCTTv1: Behavioral Change Technique Taxonomy v1.

Figure 1 outlines the entire search process. The initial screening in the Google Play Store yielded 6018 apps (screening phase: August 3, 2015 to October 6, 2015). After eliminating ineligible apps (n=4108) and removing duplicates and light versions (n=694), 1216 apps passed the first assessment with regard to mentioning a PA guideline and objective PA measurement in their description. Briefly, 2.6% (32/1216) apps mentioned a PA guideline and an objective PA measurement in their description. Four apps were no longer available for download when the testing phase started (date of availability check: February 13, 2017). After downloading and testing the remaining 28 apps, 14 did not contain any of the PA guidelines as a feature of the app, despite originally mentioning them in their description. Another 5 of 28 apps had technical problems and at least 2 different types of mobile phones failed to run the apps, and 3 did not provide objective PA measurement. In addition, 0.5% (6/1216) apps addressed PA guidelines, provided objective PA tracking, and had no technical problems—“Pacer Health/Schrittzähler & Abnehm Trainer,” “Health Mate,” “Lark Chat,” “The Walk: Fitness Tracker Game,” “Step Counter,” and “Pedometer.” Multimedia Appendix 2 provides screenshots of the selected apps.

Table 1 shows the characteristics of the 6 EIPA apps included in the content analysis. During the coding of the app content, all EIPA apps had >3.5 stars of user ratings. The highest user rating and download rate were noted for “Pacer Health/Schrittzähler & Abnehm Trainer.” “Health Mate,” “Lark Chat,” and “The Walk: Fitness Tracker Game” are English language apps, while “Pacer Health/Schrittzähler & Abnehm Trainer” is available in both English and German. “Step counter” and “Pedometer” provide Google translated versions for users. Except for “The Walk: Fitness Tracker Game,” all the apps were free of charge. For PA tracking, half of the apps (“Pedometer,” “The Walk: Fitness Tracker Game,” and “Health Mate”) used acceleration sensors, and the other half (“Step Counter,” “Pacer Health/Schrittzähler & Abnehm Trainer,” and “Lark Chat”) used a combination of accelerometer sensors and global positioning system. Of the 6 apps, 4 (ie, “Pedometer,” “Step counter,” “Pacer Health/Schrittzähler & Abnehm Trainer,” and “Health Mate”) contained the recommendation to walk 10,000 steps per day. “The Walk: Fitness Tracker Game” and “Lark Chat” were based on the PA guideline of the WHO to engage in moderate PA for at least 30 minutes per day. Furthermore, 2 apps (ie, “Health Mate” and “Pacer Health/Schrittzähler & Abnehm Trainer”) allowed users to connect their PA tracking data with other PA tracking apps such as “S Health.”

Multimedia Appendix 3 includes the definitions of the BCTs identified in the examined apps, as well as examples of how these BCTs were used in the apps.

Table 2 presents the interrater reliability between raters. The maximum agreement statistics were noted for Pedometer achieving an 89.2% prevalence-adjusted and bias-adjusted kappa and a kappa value of .42. The 2 raters had the lowest agreement in rating “Lark Chat.”

Multimedia Appendix 4 presents the type and the total number of BCTs rated for each EIPA app. Of 93 individual BCTs included in the BCTTv1, 29% (27/93) were found in the examined apps. On average, 9 (SD 5.06) were identified in apps (minimum: 4 and maximum: 18). In addition, 75% (12/16) of 16 hierarchical clusters of the BCTTv1 taxonomy were identified in the included EIPA apps. “Discrepancy between current behavior and goal standard,” “feedback on behavior,” “goal setting (behavior),” and “self-monitoring of behavior” were the most frequently included BCTs. On average, 5 (SD 3.07) hierarchical clusters were addressed in the EIPA apps. Only 2 BCTs (ie, “goals and planning” and “feedback and monitoring”) were included in all apps. The median number of BCT hierarchical clusters and BCTs included in the app was 4.5 (range 2-9) and 7.5 (range 4-18), respectively. In “Lark Chat” followed by “Health Mate,” the highest number of individual BCTs, as well as hierarchical clusters, was addressed. The maximum number of BCTs included in an EIPA app was identified for “Lark Chat” with 18 BCTs identified. The minimum number of BCTs was coded for “Pedometer,” with only 4 BCTs coded. Moreover, a maximum of 10 and a minimum of 2 hierarchical clusters were addressed in the apps included in this study. Of 16 hierarchical clusters of the taxonomy, only BCTs of 2 clusters (ie, “goals and planning” and “feedback and monitoring”) were identified in all of the apps included in this study. BCT hierarchical clusters namely “scheduled consequences” and “social support” were only identified in “Lark Chat” and “Health Mate,” respectively. Furthermore, 5 hierarchical clusters, namely “associations,” “comparison of behavior,” “comparison of outcomes,” “social support,” and “scheduled consequences” were included only once.

**Table 1:** Characteristics of physical activity and fitness apps.

Name of app	Developer(s)	Price in €	App store user rating in stars	Language	Number of app store raters	Number of downloads	Additional features
Pedometer	Ivon Liu	Free	4.0	English	470	10,000-50,000	Measures distance covered and calorie calculator
The Walk: Fitness Tracker Game	Six to Start (dev. with NHS and the UK's Department of Health)	3.39	3.7	English	1139	10,000-50,000	Google fit step tracking
Step Counter (Schrittzähler)	xstep	Free	3.5	English	363	100,000-500,000	Distance, speed, calories, heart rate, and elapsed time
Pacer (eng)/Schrittzähler & Abnehm Trainer (de)	Pacer Health	Free	4.5	English and German	330,283	10,000,000-50,000,000	Sleep tracking, nutrition (eg, water intake, vegetable intake, alcohol), pregnancy, synchronization with my fitness pal or S Health, weight
Health Mate	Withings	Free	3.6	English and German	15,222	1,000,000-5,000,000	Weight monitoring, other activities, heart rate, blood pressure, and food diary
Lark Chat	Stanford and Harvard health	Free	4.1	English	2702	50,000-100,000	Sleep tracking functionality, track other activities, such as biking, weight monitoring, and food logging

**Table 2:** Interrater reliability scores.

Name of app	Kappa	Prevalence-adjusted and bias-adjusted kappa (%)
Pedometer	.417	89.2
The Walk Fitness Tracker Game	.075	72.0
Step Counter	.332	85.0
Schrittzähler & Abnehm Trainer	.240	86.0
Health Mate	.285	72.0
Lark Chat	.031	52.6

## Discussion

### Principal Findings

This study aimed to identify EIPA apps and BCTs employed in these apps. The results revealed that <1% of the examined 1216 apps mentioned PA guidelines, an objective PA measurement, and worked properly. Regarding BCTs included in those EIPA apps, approximately one-third of the BCTs outlined in the BCTTv1 were used. Moreover, BCTs of 75% (12/16) of the 16 hierarchical clusters of the taxonomy were identified, the overarching clusters “goals and planning” and “feedback and monitoring” were included in all of them.

### Comparison With Prior Studies

In comparison to our results, a review of 2400 PA apps conducted by Knight et al reported that none of the examined apps were based on evidence-based guidelines for aerobic PA, and only approximately 2% (8/379) of 379 apps deemed eligible were implementing evidence-based guidelines for resistance training [14]. Modave et al reviewed 30 popular PA apps and reported that only 3 apps reflected parts of the guidelines set forth by the ACSM [13]. Our search may have yielded different results because we searched the German Google Play Store, our search strategy was different, and the search was conducted during a later point in time. The updated versions of apps or newly developed apps may increasingly address PA guidelines and incorporate a larger number of BCTs.

Taking a somewhat similar approach to our study, Direito et al downloaded the top-20 paid and top-20 free PA and dietary behavior apps from the New Zealand Apple App Store Health and Fitness category and coded each app for the presence or absence of BCTs [25]. They coded approximately 20% of BCTs from the BCTTv1 compared with 30% in our content analysis. Similar to our study, they found, on average, 8 BCTs (range 2-18) in these apps. Furthermore, they found that paid apps included more BCTs [25]. Whether there was a statistically significant difference between paid apps and those free of charge regarding the number of BCTs was not investigated in this study. The most commonly identified BCTs in Direito et al’s study were “provide instruction,” “set graded tasks,” and “prompt self-monitoring” [25].This is contrary to the BCTs most commonly found in our study (ie, “discrepancy between current behavior and goal standard,” “feedback on behavior,” “goal setting (behavior),” and “self-monitoring of behavior”). However, this discrepancy of results may be attributed to the differences in the scope of the 2 searches. Direito et al only examined the most downloaded apps [25]. Results comparable to our results were obtained in an extensive content analysis conducted by Middelweerd et al based on an earlier version of the taxonomy [19].

In another review of weight management apps, Bardus et al rated the app quality and content of the most popular health and fitness apps on Google Play and iTunes to determine the number of BCTs included [26]. In their study, 10 techniques were identified per app (range 1-17) and approximately 37% of BCTs from the BCTTv1 were applied with “goal setting” and “self-monitoring” among the most frequently identified. In addition, Bardus et al found that the number of BCTs included correlated with the app quality and the number of different technical features of apps [26]. Unfortunately, the app quality was not assessed in our study. Furthermore, which combination of BCTs is most effective in changing PA behavior and whether there is an association between including a higher number of BCTs in PA apps and changes in PA remain questions to be addressed in future studies. There is some indication of a review of studies examining the effects of interventions targeting healthy eating and PA that a combination of self-monitoring with at least one other technique derived from control theory was more effective in promoting behavior change than other single-technique interventions [27]. In addition, different BCTs may be effective for promoting short-term versus long-term changes in health behavior. Samdal et al demonstrated that “goal setting” and “self-monitoring of behavior” were associated with both short-term and long-term changes in healthy eating and PA in overweight and obese adults, whereas several other BCTs (eg, “goal setting of outcome,” “feedback on outcome of behavior,” “implementing graded tasks,” “adding objects to the environment,” such as step counters) predicted behavior change in the long term [28].

User ratings of >3.5 stars were noted for all EIPA apps examined in this study. The relationship between user rating and the number of BCTs included in apps is, however, still unclear and requires further investigation. In addition, it remains unknown whether EIPA apps include more BCTs than generic PA apps and therefore receive higher user ratings. The findings of a previous study indicated that user ratings positively correlate with the number of features included in PA apps [29]. However, a subgroup analysis on Google Play versus iTunes apps performed by Mollee et al to evaluate their potential for increasing PA yielded contradictory associations of user ratings and the number of features for Android versus iTunes PA apps [29]. User ratings were not associated with the number of features for Android PA apps [29], while in another study, a 15% increment of user ratings was noted for each additional BCT included in iTunes PA apps [30]. However, these associations may be misleading because user ratings can be easily manipulated. It has previously been reported that app developers can recruit users with as low as US $5 to negatively review or badly rate apps developed by their competitors [31,32]. Nevertheless, future studies should explore the interrelationships between user ratings, number of BCTs, additional features, price, PA measurement accuracy, and effectiveness of apps for PA promotion further. In addition, new tools, such as the Mobile App Rating Scale, may assist researchers in determining the quality of apps [33]. For example, differences in the accuracy of measuring distance were noted by Pobiruchin et al [34]. Hence, PA measurement accuracy of PA apps regarding objective indicators (eg, distance covered, steps counted, and timing of exercises) needs further evaluation and calibration, using gold standards.

### Limitations and Strengths

This study has several limitations. First, the study was limited to Android PA apps. The inclusion of iTunes PA apps might have produced different results. Second, one problem encountered during the search was that the screening process took a long time because the search was not limited to the most popular or downloaded PA apps. Some of the apps were no longer available, or updates were available when the testing phase was reached. The restricted search strategies followed in the studies outlined above may have prevented our search from being outdated by the time the content was coded in detail. However, we ensured that the apps tested for 2 weeks were still available at the beginning of the testing phase. In some cases, there were updated versions, which had been further developed by the same or a different company. Another limitation was that the maximum number of apps available per search term was limited to 250 apps and the underlying algorithm for this limitation was unclear. Considering the rapid development and release of PA apps, a new search would produce very different findings. In addition, it remains unclear whether searches in other regions of Europe or the world would produce similar results. Therefore, the generalizability of the results is limited. Another issue encountered during the search was that app descriptions were in many cases different from the functions offered in the apps, resulting in a retrospective exclusion of apps during the testing phase. We may have excluded apps that did not explicitly mention PA guidelines in their descriptions but were guideline-informed. However, the strength of our study was the relatively high interrater reliability for identifying BCTs in the final selection of apps suggesting that raters were well versed in the use of the taxonomy.

In sum, our results are in line with existing research indicating that only a limited number of BCTs is currently included in such interventions despite growing evidence suggesting that the effectiveness of digital health interventions can be enhanced by incorporating BCTs [27,28]. In addition, the existing evidence suggests that the theoretical constructs of BCTs are only rarely considered during app development [18,19,35,36]. Hence, there appears to be a need for collaboration between PA app developers and public health, health promotion, and behavior change experts [35,37].

### Conclusions

To conclude, this study indicates that despite the availability of several thousand PA apps for Android platforms, very few of them are evidence informed and simultaneously provide objective PA measurement. In addition, only a few of them incorporate a large number of BCTs. Future apps should address evidence-based PA guidelines and a greater scope of BCTs to further increase their potential impact for PA promotion in the general population. Furthermore, it is important that researchers make recommendations regarding the use of EIPA apps in the general population or advise health insurances in selecting and disseminating the EIPA apps identified in this study to insurance holders as opposed to representatives of entities with commercial interests. The widespread use of EIPA apps may boost other population-based strategies for PA promotion for primary prevention in Germany, which are currently ongoing as a result of the Preventive Health Care Act.

## Appendix

Multimedia Appendix 1 Results of the testing phase (n=32 apps).

Multimedia Appendix 2 Screenshots of the included apps.

Multimedia Appendix 3 Definitions of behavioral change techniques (BCTs) addressed in the included evidence-informed physical activity apps and examples for application of BCTs in apps.

Multimedia Appendix 4 Individual behavior change techniques and hierarchical clusters addressed in the evidence-informed physical activity apps included in the content analysis.

## Abbreviations

ACSM: American College of Sports Medicine
BCT: Behavior change technique
BCTTv1: Behavioral Change Technique Taxonomy v1
eHealth: electronic health
EIPA: Evidence-informed physical activity
mHealth: mobile health
PA: physical activity
WHO: World Health Organization
