# Supplementary material for: Identifying Evidence-Informed Physical Activity Apps: Content Analysis
Source: JMIR Mhealth Uhealth. 2018 Dec 18;6(12):e10314. doi: 10.2196/10314 (PMC6315275; doi:10.2196/10314)
Supplement: Multimedia Appendix 2 [file mhealth_v6i12e10314_app2.pdf]

Health Mate

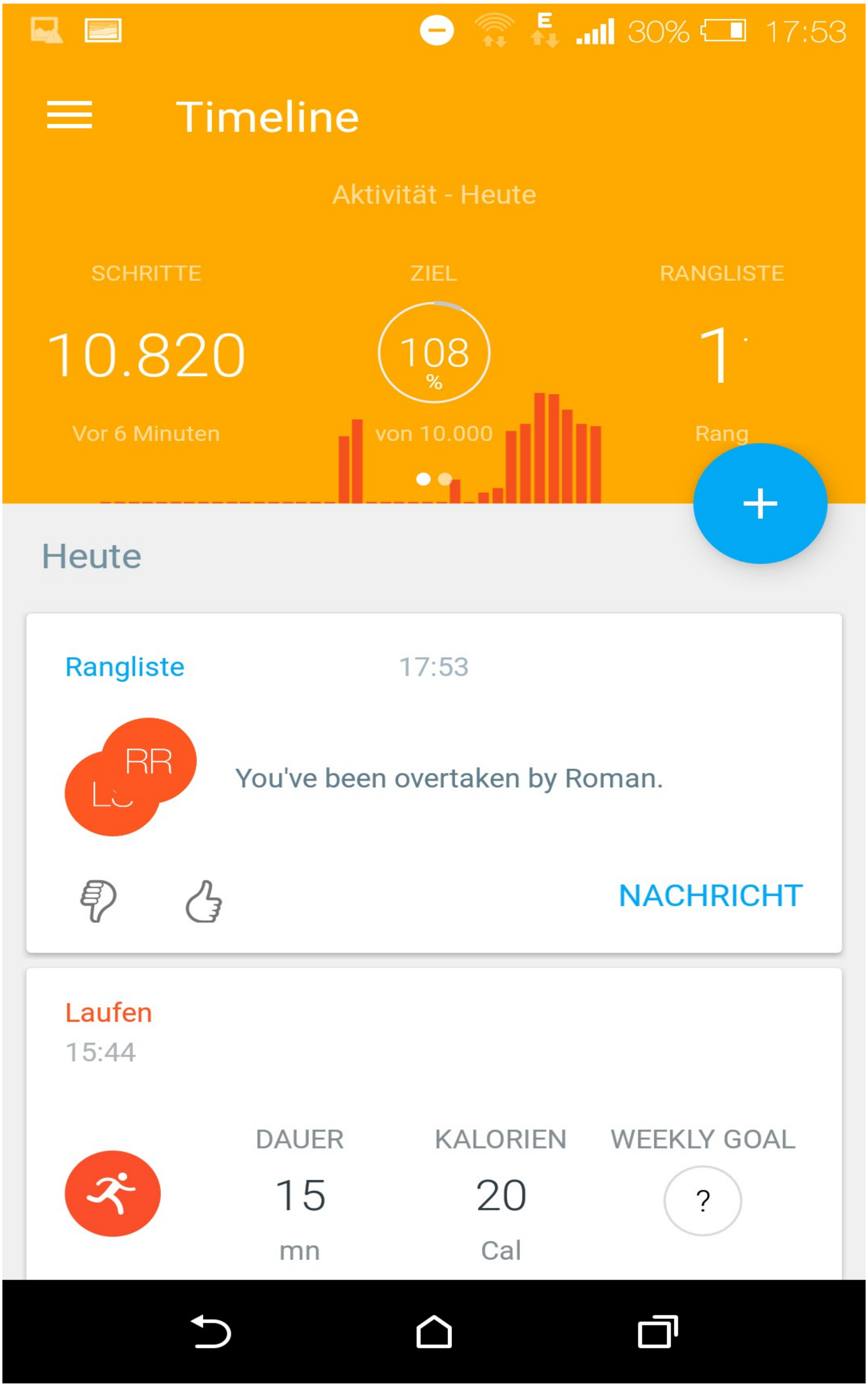

30% 17:53
Timeline
Aktivität - Heute
SCHRITTE
ZIEL
RANGLISTE
10.820
108 %
1
Vor 6 Minuten
von 10.000
Rang
Heute
Rangliste
17:53
RR
You've been overtaken by Roman.
NACHRICHT
Laufen
15:44
DAUER
KALORIEN
WEEKLY GOAL
15
mn
20
Cal
?

Lark Chat

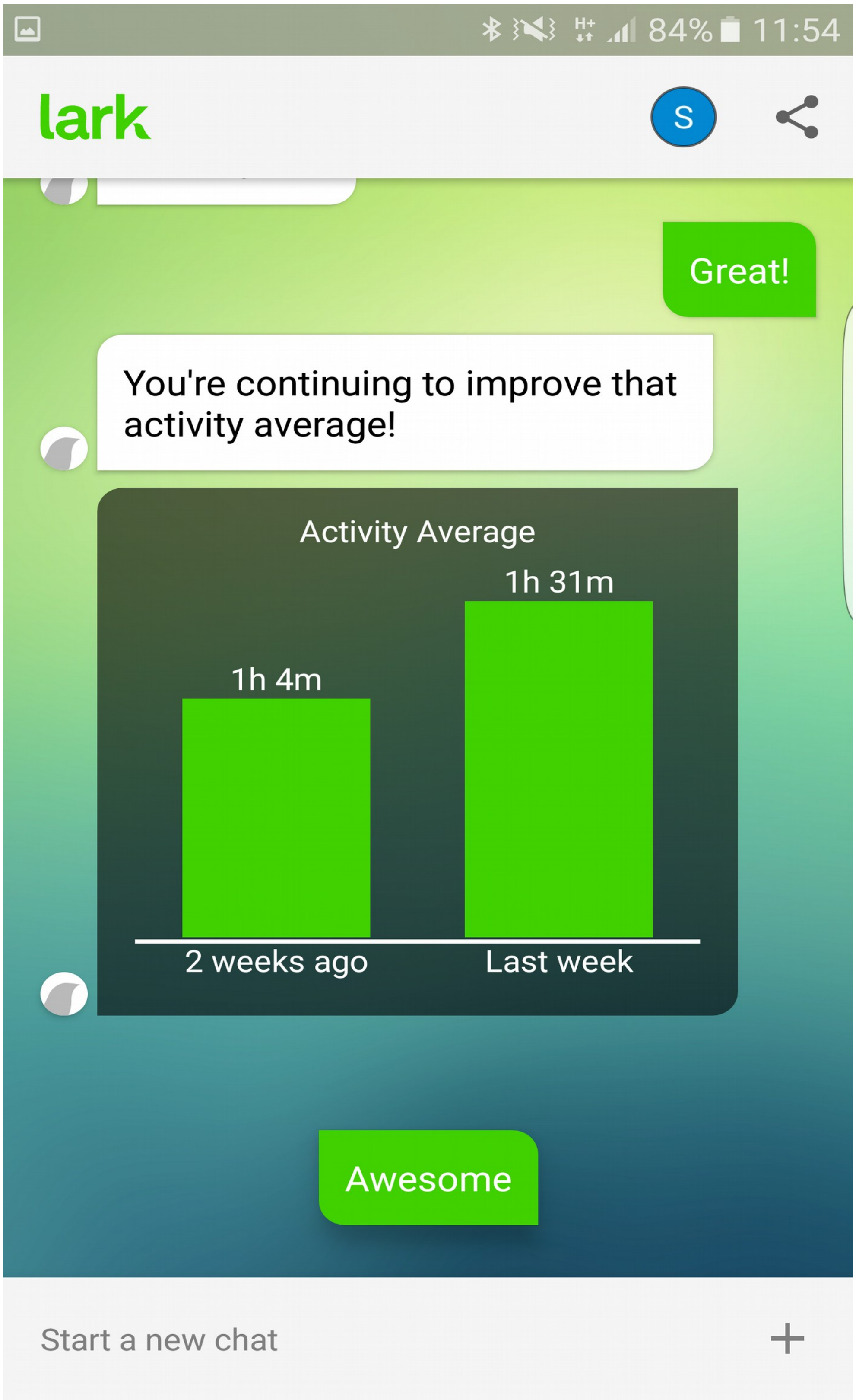
84% 11:54
lark
S
Great!
You're continuing to improve that activity average!
Activity Average
1h 31m
1h 4m
2 weeks ago
Last week
Awesome
Start a new chat

Schrittzähler & Abnehm Trainer

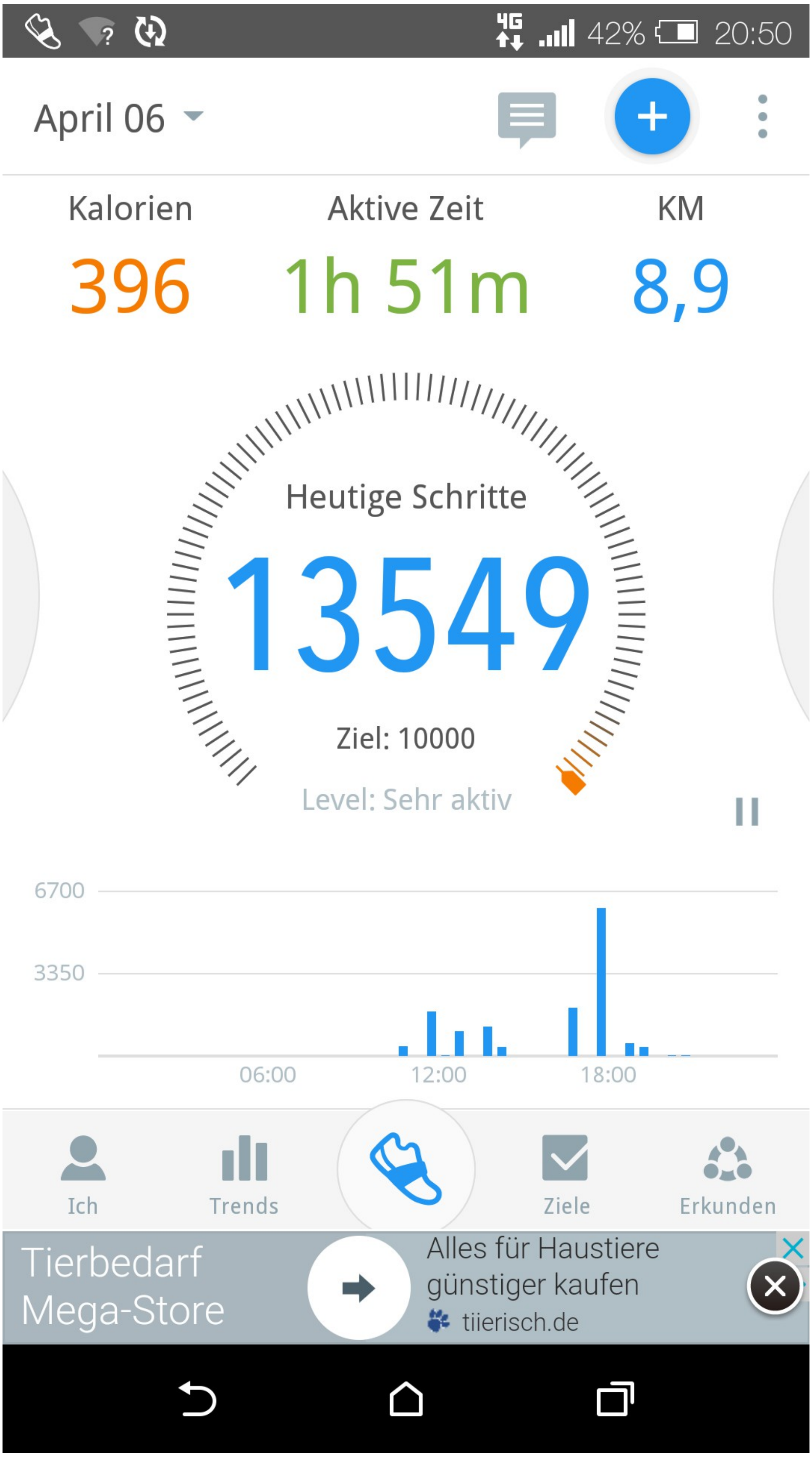

The Walk Fitness Tracker Game

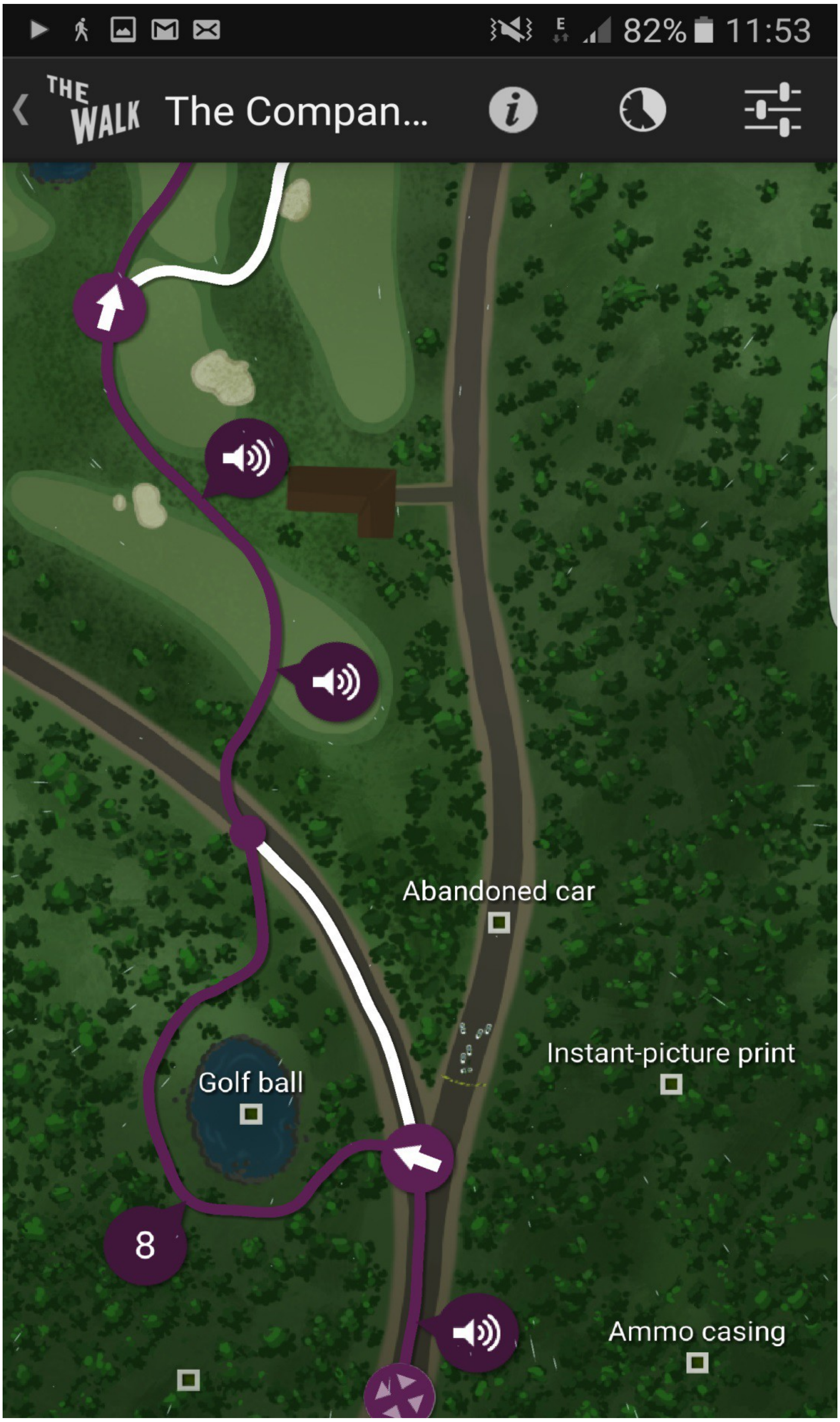

Step Counter

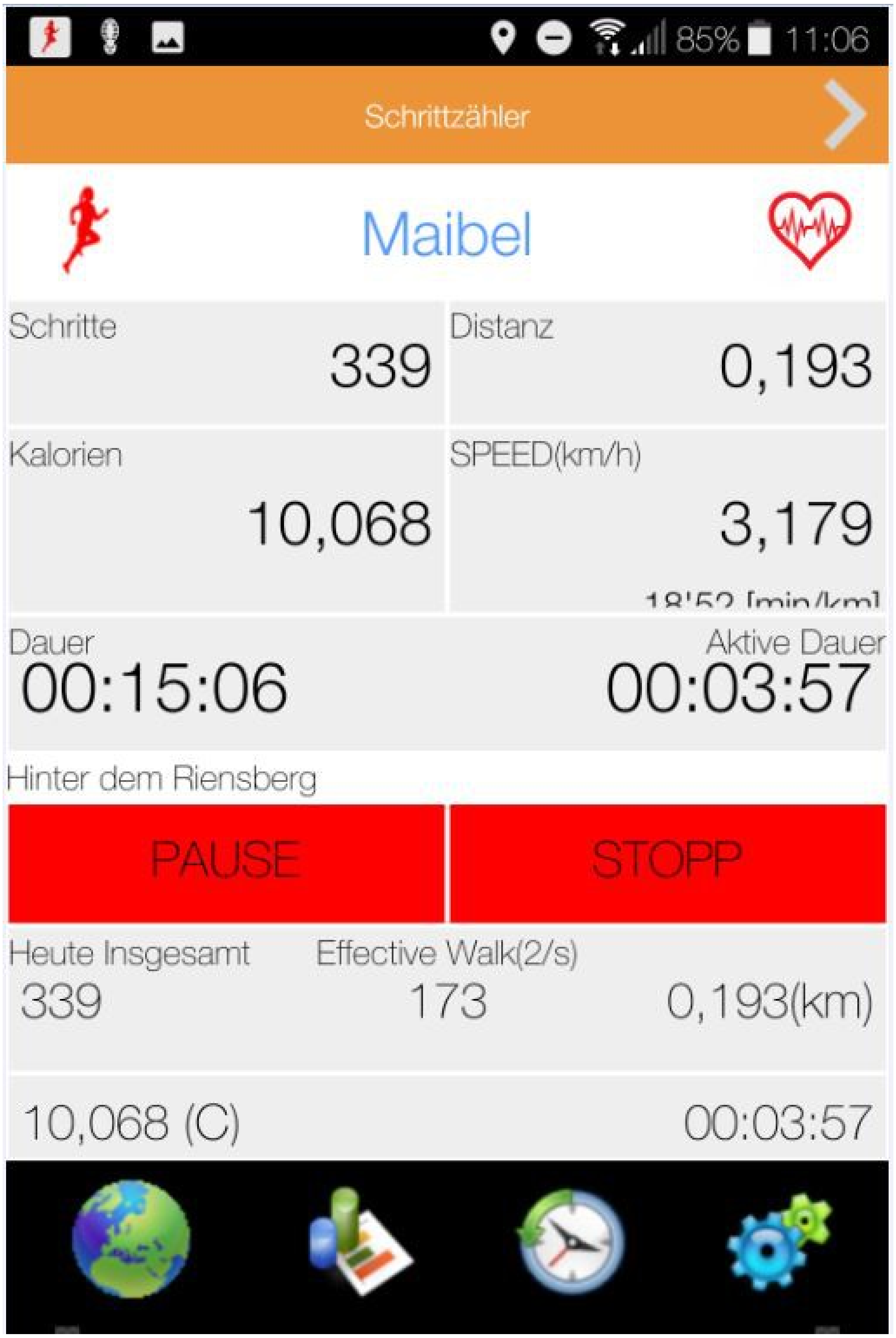

Pedometer

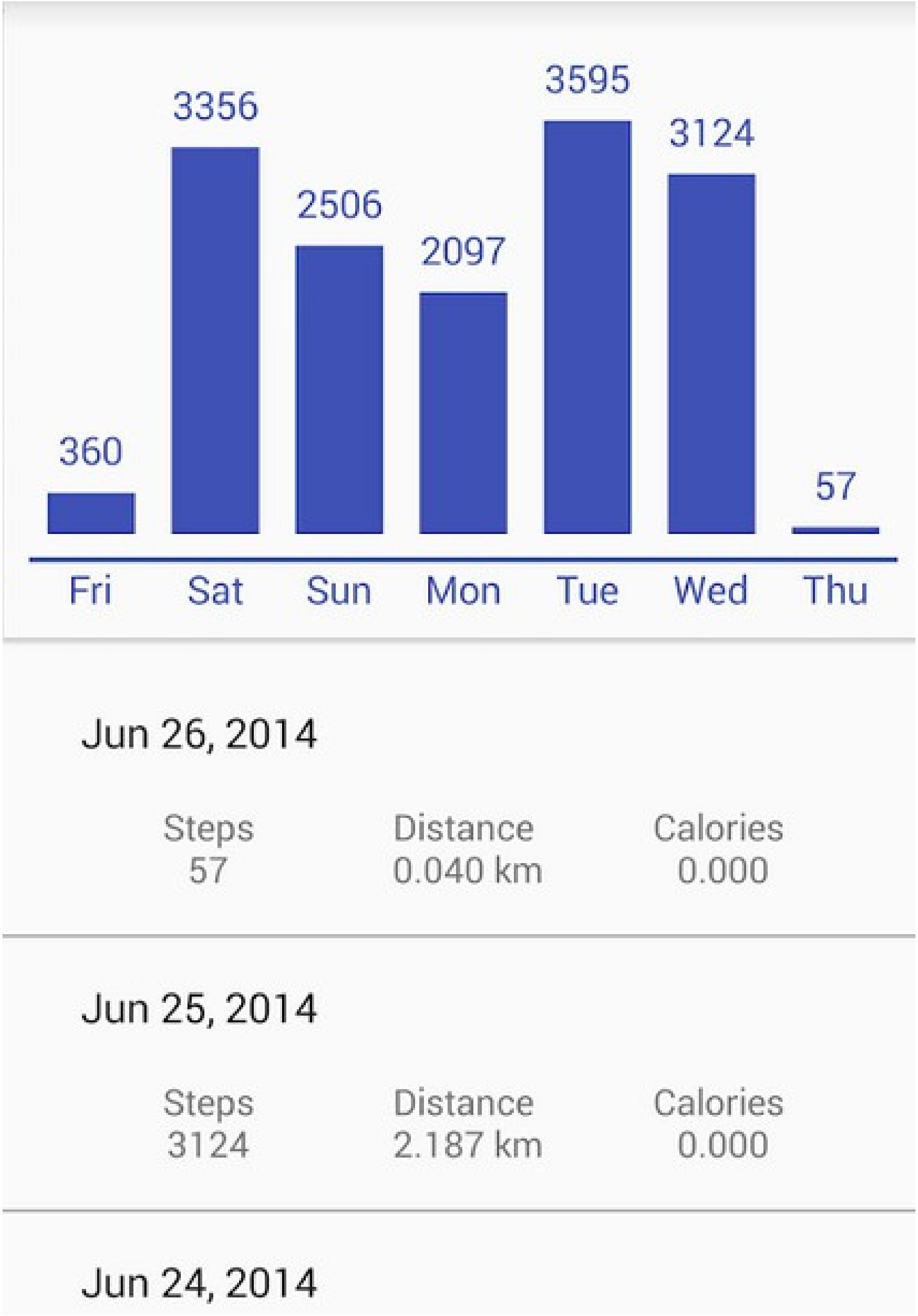

3356
3595
3124
2506
2097
360
57
Fri
Sat
Sun
Mon
Tue
Wed
Thu
Jun 26, 2014
Steps
57
Distance
0.040 km
Calories
0.000
Jun 25, 2014
Steps
3124
Distance
2.187 km
Calories
0.000
Jun 24, 2014
