# Supplementary material for: Identifying Evidence-Informed Physical Activity Apps: Content Analysis
Source: JMIR Mhealth Uhealth. 2018 Dec 18;6(12):e10314. doi: 10.2196/10314 (PMC6315275; doi:10.2196/10314)
Supplement: Multimedia Appendix 3 [file mhealth_v6i12e10314_app3.pdf]

Multimedia Appendix 3. Definitions of behavioral change techniques (BCTs) addressed in the included evidence-informed physical activity apps and examples for application of BCTs in apps.

BCT<br>(Number indicating hierarchical cluster and respective BCT, Michie et al, 2013)	Hierarchical cluster<br>(Michie et al, 2013)	Definition (Michie et al, 2013)	Example
Goal setting (behavior) [1.1]	Goals and planning	Set or agree on a goal defined in terms of the behavior to be achieved	Set the goal of walking 10,000 steps daily
Problem solving/coping planning [1.2]	Goals and planning	Analyze, or prompt the person to analyze factors influencing the behavior and generate or select strategies that include overcoming barriers and/or increasing facilitators	Advice to get back to the usual PA/exercise routine after not being physically active because of day-to-day stress(ors)/having a busy life; contemplate reasons for wanting to be physically active (healthy choices as a privilege)
Goal setting (outcome) [1.3]	Goals and planning	Set or agree on a goal defined in terms of a positive outcome of wanted behavior	Set a weight loss goal as an outcome of increased PA
Action planning [1.4]	Goals and planning	Prompt detailed planning of performance of the behavior (must include at least one of context, frequency, duration, and intensity). Context may be environmental (physical or social) or internal (physical, emotional or cognitive)	Provision of diary function to document time and duration of PA (and take notes regarding context, e.g., for running, biking, and workout)
Review behavior goal(s) [1.5]	Goals and planning	Review behavior goal(s) jointly with the person and consider modifying goal(s) or behavior change strategy in light of achievement. This may lead to re-setting the same goal, a small change in that goal or setting a	10,000 steps is the default behavior goal; goals can be adjusted after reaching previously set goals

		new goal instead of (or in addition to) the first, or no change	
Discrepancy between current behavior and goal standard [1.6]	Goals and planning	Draw attention to discrepancies between a person's current behavior and the person's previously set outcome goals, behavioral goals or action plans	Displays how many steps of the target (e.g., 10,000 steps) have already been achieved
Feedback on behavior [2.2]	Feedback and monitoring	Monitor and provide informative or evaluative feedback on performance of the behavior	A log or graph that displays the user's performance with regard to walking 10,000 steps and praise/affirmative comments for walking a certain number of steps
Self-monitoring of behavior [2.3]	Feedback and monitoring	Establish a method for the person to monitor and record their behavior(s) as part of a behavior change strategy	Display of number or graph of steps walked
Self-monitoring of outcome(s) of behavior [2.4]	Feedback and monitoring	Establish a method for the person to monitor and record the outcome(s) of their behavior as part of a behavior change strategy	Option to document individual weight over a certain period of time
Feedback on outcome(s) of behavior [2.7]	Feedback and monitoring	Monitor and provide feedback on the outcome of performance of the behavior	A log or graph that displays the user's weight loss (trend) over a certain period of time (e.g., −0.2 kg)
Social support (general) [3.1]	Social support	Advise on, arrange or provide social support or non-contingent praise or reward for performance of the behavior. It includes encouragement and counselling, but only when it is directed at the behavior	"Adding friends" function, provision of the possibility to chat and support each other
Information about antecedents [4.2]	Shaping knowledge	Provide information about antecedents that reliably predict performance of the behavior	Prompt reflection of how a person was able to motivate themselves to perform extra PA during a particular day
Reattribution [4.3]	Shaping knowledge	Elicit perceived causes of behavior and suggest alternative explanations	Reflection of own behavior to find actual reasons for more or less PA which may help the person to stay

		(e.g., external or internal and stable or unstable)	physically active
Information about health consequences [5.1]	Shaping knowledge	Provide information (e.g., written, verbal, visual) about health consequences of performing the behavior	Displays of written information about positive health consequences of increased PA (e.g., decreasing risk for cardiovascular diseases and better health-related quality of life)
Information about emotional consequences [5.6]	Natural consequences	Provide information (e.g., written, verbal, visual) about emotional consequences of performing the behavior	Displays written information about improved mood/affect and concentration as a result of increased PA
Social comparison [6.2]	Comparison of behavior	Draw attention to others' performance to allow comparison with the person's own performance	Comparison of own performance with that of others and provision of information on ranking of individuals
Prompts/ cues [7.1]	Associations	Introduce or define environmental or social stimulus with the purpose of prompting or cueing the behavior. The prompt or cue would normally occur at the time or place of performance	Reminder when person was inactive for >30 minutes
Behavioral rehearsal/practice [8.1]	Repetition and substitution	Prompt practice or rehearsal of the performance of the behavior one or more times in a context or at a time when the performance may not be necessary in order to increase habit and skill	Affirmation when PA is performed during an unusual time (e.g., in the mornings)
Behavior substitution [8.2]	Repetition and substitution	Prompt substitution of the unwanted behavior with a wanted or neutral behavior	Comment not to fall into couch potato status again and to stick with previous days during which person was more physically active; suggestion to perform self-nurturing activities to get back on track with PA routine
Habit formation [8.3]	Repetition and substitution	Prompt rehearsal and repetition of the behavior in the same context repeatedly so that the context	Proposal to repeatedly go on short walks; encouragement to repeat the behavior; advise that consistency is a

		elicits the behavior	predictor of continued PA
Graded tasks [8.7]	Repetition and substitution	Set easy-to-perform tasks, making them increasingly difficult, but achievable, until behavior is performed	Every new PA level has a longer duration and requires longer bouts of walking over longer distances
Persuasive argument [9.1]	Comparison of outcomes	Present verbal or visual communication from a credible source in favor of or against the behavior	Emphasis on the recommendation of the WHO to walk 10,000 steps/day
Non-specific reward [10.3]	Reward and threats	Arrange delivery of a reward if and only if there has been effort and/or progress in performing the behavior	Segments of stories were provided as a reward for reaching earlier goals
Social reward [10.4]	Reward and threats	Arrange verbal or non-verbal reward if and only if there has been effort and/or progress in performing the behavior	User is being congratulated to his/her success in achieving the behavior goal
Reward approximation [14.4]	Scheduled consequences	Arrange for reward following any approximation to the target behavior, gradually rewarding only performance closer to the wanted behavior	Rewards are only provided when increase in PA is greater than during the days prior to the actual day
Verbal persuasion about capability [15.1]	Self-belief	Tell the person that they can successfully perform the wanted behavior, arguing against self-doubts and asserting that they can and will succeed	Affirmation that person is reaching the behavior goal with their preferred PA (e.g., with bike rides)
Focus on past success [15.3]	Self-belief	Advise to think about or list previous successes in performing the behavior (or parts of it)	Display average activity graph and comment on recent PA in relation to previous days during which more PA was performed
