# Supplementary material for: Identifying Evidence-Informed Physical Activity Apps: Content Analysis
Source: JMIR Mhealth Uhealth. 2018 Dec 18;6(12):e10314. doi: 10.2196/10314 (PMC6315275; doi:10.2196/10314)
Supplement: Multimedia Appendix 4 [file mhealth_v6i12e10314_app4.pdf]

Multimedia Appendix 1. Individual behavior change techniques and hierarchical clusters addressed in the evidence-informed physical activity apps included in the content analysis.

		Name of the evidence-informed physical activity app[a]						Total
BCT (Number indicating the cluster and respective BCT, Michie et al, 2013)	Hierarchical cluster (Michie et al, 2013)	Pedometer	The Walk: Fitness Tracker Game	Step Counter	Schrittzähler & Abnehm Trainer	Health Mate	Lark Chat	
1. Discrepancy between current behavior and goal standard [1.6]	Goals and planning	✔	✔	✔	✔	✔	✔	6
2. Feedback on behavior [2.2]	Feedback and monitoring	✔	✔	✔	✔	✔	✔	6
3. Goal setting (behavior) [1.1]	Goals and planning	✔	✔	✔	✔	✔		5
4. Self-monitoring of behavior [2.3]	Feedback and monitoring	✔		✔	✔	✔	✔	5
5. Prompts/cues [7.1]	Associations		✔	✔		✔	✔	4
6. Goal setting (Outcome) [1.3]	Goals and planning			✔	✔			2
7. Self-monitoring of outcome(s) of behavior [2.4]	Feedback and monitoring			✔	✔		✔	3
8. Information about health consequences [5.1]	Natural consequences					✔	✔	2
9. Social comparison [6.2]	Comparison of behavior				✔	✔		2
10. Social reward [10.4]	Reward and threat					✔	✔	2
11. Persuasive Argument	Comparison of outcomes				✔		✔	2

[9.1]								
12. Problem solving/coping planning [1.2]	Goals and planning						✔	1
13. Action planning [1.4.]	Goals and planning						✔	1
14. Review behavior goal(s) [1.5]	Goals and planning					✔		1
15. Feedback on outcome(s) of behavior [2.7]	Feedback and monitoring				✔			1
16. Social support (general) [3.1]	Social support					✔		1
17. Information about antecedents [4.2]	Shaping knowledge						✔	1
18. Reattribution [4.3]	Shaping knowledge						✔	1
19. Information about emotional consequences [5.6]	Natural consequences					✔		1
20. Behavioral rehearsal/practice [8.1]	Repetition and substitution						✔	1
21. Behavior substitution [8.2]	Repetition and substitution						✔	1
22. Habit formation [8.3]	Repetition and substitution						✔	1
23. Graded tasks [8.7]	Repetition and substitution		✔					1
24. Non-specific reward [10.3]	Reward and threat		✔					1
25. Reward	Scheduled consequences						✔	1

approximation [14.4]								
26. Verbal persuasion about capability [15.1]	Self-belief						✔	1
27. Focus on past success [15.3]	Self-belief						✔	1
Total number of BCTs included in the app		4	6	6	9	11	18	

[a]BCTs not addressed in apps: Review of outcome goal(s) [1.7], Behavioral contract [1.8], Commitment [1.9], Monitoring of behavior by others without feedback [2.1], Monitoring of outcome(s) of behavior by others without feedback [2.5], Biofeedback [2.6], Social support (practical) [3.2], Social support (emotional) [3.3], Instruction on how to perform a behavior [4.1], Behavioral experiments [4.4], Salience of consequences [5.2], Information about social and environmental consequences [5.3], Self-assessment of affective consequences/Monitoring of emotional consequences [5.4], Anticipated regret [5.5], Modeling/Demonstration of the behavior [6.1], Information of other's approval [6.3], Discriminative (learned cue)/Cue signaling reward [7.2], Fading/Reduce prompts/cues [7.3], Time out/Remove access to the reward [7.4], Escape learning/Remove aversive stimulus [7.5], Satiation [7.6], Exposure [7.7], Classical conditioning/Associative learning [7.8], Habit reversal [8.4], Overcorrection [8.5], Generalization of a target behavior [8.6], Pros and cons [9.2], Comparative imagining of future outcomes [9.3], Material incentive (behavior) [10.1], Material reward (behavior) [10.2], Social incentive [10.5], Non-specific incentive [10.6], Self-incentive [10.7], Incentive (outcome) [10.8], Self-reward [10.9], Reward (outcome) [10.10], Threat/Future punishment [10.11], Pharmacological support [11.1], Regulate/Reduce negative emotions [11.2], Conserving mental resources [11.3], Paradoxical instructions [11.4], Restructuring the physical environment [12.1], Restructuring the social environment [12.2], Avoidance/changing exposure to cues for the behavior [12.3], Distraction [12.4], Adding objects to the environment [12.5], Body changes [12.6], Identification of self as role model [13.1], Reframing/framing [13.2], Cognitive dissonance/incompatible beliefs [13.3], Self-affirmation/Valued self-identity [13.4], Identity associated with changed behavior [13.5], Behavior/response cost [14.1], Punishment [14.2], Extinction/Remove reward [14.3], Chaining/Rewarding completion [14.5], Discrimination training/Situation-specific reward [14.6], Counter-conditioning/Reward incompatible behavior [14.7], Differential reinforcement/Reward alternative behavior [14.8], Thinning/Reduce reward frequency [14.9], Negative Reinforcement/Remove punishment [14.10], Mental rehearsal of successful performance [15.2], Self-talk [15.4], Covert sensitization/Imaginary punishment [16.1], Covert conditioning/Imaginary reward [16.2], Vicarious reinforcement/Vicarious consequences [16.3]
